# Reduction in Ocular Hypotensive Eyedrops by Ab Interno Trabeculotomy Improves Not Only Ocular Surface Condition But Also Quality of Vision

**DOI:** 10.1155/2018/8165476

**Published:** 2018-06-21

**Authors:** Kenji Kashiwagi, Mio Matsubara

**Affiliations:** Department of Ophthalmology, University of Yamanashi Faculty of Medicine, Chuo, Yamanashi, Japan

## Abstract

**Purpose:**

To investigate the effect of ab interno trabeculotomy using the Trabectome surgical system on tear film stability and functional visual acuity (FVA).

**Patients and Methods:**

Adult glaucoma patients who underwent Trabectome surgery alone or Trabectome surgery combined with phacoemulsification with intraocular lens insertion were included in this study. Corneal epithelial defects, tear film breakup time (TBUT), tear meniscus height, tear film spreading grade, tear interferometry grade, and FVA were assessed before and after surgery in addition to routine ophthalmic examinations. Changes in ocular surface conditions and visual acuity as a result of the Trabectome surgery were investigated.

**Results:**

Thirty eyes of 22 patients with a mean age of 72.2 ± 7.9 years, including 8 males and 14 females, were enrolled. The Trabectome surgery significantly reduced the intraocular pressure (IOP) from 20.3 ± 5.2 to 15.0 ± 3.3 mmHg (*P* < 0.001) and the number of different types of ocular hypotensive eyedrops used from 3.2 ± 0.7 to 1.1 ± 0.7 types (*P* < 0.001). The surgery significantly improved corneal epithelial defects, the tear spreading grade, the tear interferometry grade, and FVA. The surgery also improved the visual maintenance ratio among all enrolled patients, including those who underwent Trabectome surgery only.

**Conclusion:**

Trabectome surgery may be beneficial not only for IOP reduction but also for improving ocular surface conditions and FVA.

## 1. Introduction

Glaucoma is a disease that affects individuals across the lifespan, and many patients are required to use ocular hypotensive ophthalmic solutions. In previous reports, glaucoma patients used approximately two ophthalmic solutions [1], and ocular surface damage was common. Rossi et al. reported that patients with topically treated glaucoma presented with dry eye syndrome more often than the control group [2], Leung et al. reported that approximately 60% of glaucoma patients had symptoms of dry eye and that the number of benzalkonium chloride- (BAC-) containing eyedrops was associated with the severity of ocular surface disease [3].

Because tear film function requires a regular and smooth ocular surface with a stable tear film to form clear visual images, ocular surface damage causes several symptoms including red eye, photophobia, eye itching, blurring, and other discomforts that sometimes deteriorate a patient's quality of life [2, 4].-

A smaller amount of ocular hypotensive ophthalmic solution may result in better ocular surface condition. Trabeculectomy with mitomycin C is major glaucoma surgery and successfully reduces the number of ocular hypotensive eyedrops, but previous papers reported that trabeculectomy with mitomycin C deteriorated ocular surface conditions due to adverse effects of mitomycin D or deterioration of tear film replacement [5]. The Trabectome system (Neomedix, Tustin, CA) is used for performing trabeculotomy via an internal approach. This procedure successfully reduces the number of topical medications in addition to the intraocular pressure (IOP) [6], which may contribute to ocular surface stability. The Trabectome system does not use antimetabolites, and there is less irregularity of tear film formation. We therefore hypothesized that the Trabectome system may be beneficial to the ocular surface.

A functional visual acuity (FVA) test can evaluate continuous visual acuity changes over time, which may be better for evaluating the quality of vision than a conventional visual acuity test. The methodology of FVA testing has evolved since it first emerged as a promising technology to evaluate changes in visual acuity over time [7, 8]. Some ocular conditions have been reported to influence FVA. Goto et al. reported that dry eye decreased FVA among patients who had normal conventional visual acuity [8, 9]. FVA is significantly reflected in the tear functions and ocular surface status of the eye. The FVA test could help detect the masked impairment of visual function among patients in patients who complain of decreased visual acuity despite having normal visual acuity measurements using conventional testing.

In this study, we investigated the effects of ab interno trabeculotomy using the Trabectome system on tear film stability in terms of superficial punctate keratitis, tear film breakup time (TBUT), height of the tear meniscus, and tear film lipid layer interference patterns as well as visual acuity, which was assessed using a conventional visual acuity test and FVA test.

## 2. Patients and Methods

This study was performed as a prospective cohort study, approved by the Ethics Committee of the University of Yamanashi and conducted in accordance with the Helsinki Declaration and the Ethical Guidelines for Medical and Health Research Involving Human Subjects of the Japanese Ministry of Health, Labor and Welfare. All participants provided written informed consent.

### 2.1. Inclusion and Exclusion Criteria

Among all glaucoma patients who underwent Trabectome surgery alone or combined surgery with phacoemulsification and intraocular lens insertion from December 2016 to April 2017, subjects who satisfied the following criteria were enrolled: adult age, a history of use of ocular hypotensive eyedrops for one year or longer, provided written informed consent, no complications during the surgery, best-corrected visual acuity of 20/100 or better before and after the surgery, and completion of the study protocol. The exclusion criteria were as follows: a threat of central visual field loss, specifically, two or more of the four central measured points on the Humphrey field analyzer (HFA) (Carl Zeiss Meditec, Dublin, CA) 10-2 program showing less than 10 dB of sensitivity; any diseases that affect tear film stability, including Sjogren's syndrome; any diseases that affect visual acuity, except for cataracts; astigmatism with ≥1.5 diopters of corneal curvature; a history of intraocular surgery, except cataract surgery performed same or more than one year prior to the study; and administration of routine ophthalmic solutions, except ocular hypotensive solutions and hyaluronic acid solutions.

### 2.2. General Ophthalmic Examinations

The following examinations were performed before and after surgery: best-corrected visual acuity, refractive error measurement, slit-lamp examination, fundus examination, and IOP measurement using a Goldmann applanation tonometer. Visual field test using the HFA10-2 and 24-2 programs, and optic nerve evaluation using an optical coherence tomography CIRRUS HD-OCT (Carl Zeiss Meditec, Inc., Dublin, CA) were performed within three months before surgery.

### 2.3. Ocular Surface Evaluation

To evaluate the ocular surface, we employed the following tests:

### 2.4. Tear Film Breakup Time Examination

Trained medical staff evaluated the TBUT using a DR-1*α* (Kowa, Nagoya, Japan) to assess tear film stability in a blinded protocol. The DR-1*α* examination was repeated two times, and recorded video images were subject to analysis. A staff member evaluated the TBUT, which was calculated as the mean measurement of two trials.

### 2.5. Tear Meniscus Evaluation

An expert ophthalmologist (K.K.) evaluated the tear meniscus height using a tear turnover assessment test. The tear meniscus height was evaluated by adjusting the vertical length of a slit beam on the tear meniscus at the center of the lower lid, and the values were read from the slit-lamp scale [10].

### 2.6. Corneal Erosion Evaluation

Corneal erosion was evaluated by introducing a wetted 0.7 mg fluorescein sodium ophthalmic strip (FLUORES Ocular Examination Test Paper, Ayumi Pharmaceutical Ltd., Tokyo, Japan) into the inferior fornix. The graded corneal erosion system [11] was adopted as the ocular surface damage assessment. Briefly, two parameters of corneal erosion, area and density, were graded on a scale ranging from A0 to A3 and from D0 to D3, respectively.

### 2.7. Tear Interferometry Test

The tear interferometry test was conducted using DR-1*α*. Detailed information has been described elsewhere [13]. In brief, this instrument observes the specular reflected light from the tear surface of a circular area 2 mm in diameter of the central cornea. Lipid layer interference images were recorded soon after a complete blink. The interference patterns of the tear film lipid layer were analyzed and graded from I to V according to the Yokoi standards of grading [12, 13].

Grade I or grade II was considered normal, whereas grade III or above was considered abnormal. In addition, the spreading of a tear film lipid layer was evaluated using 4 grades as follows: grade 1: the tear film smoothly spread across all of the corneal area; grade 2: the tear film slowly spread across more than half of the corneal area; grade 3: the tear film slowly spread across less than half of the corneal area; and grade 4: the tear film never spread on the cornea. Representative images of the lipid layer interference pattern and the tear film spreading patterns are shown in Supplementary Figures 1 and 2.

### 2.8. Functional Visual Acuity Test

We performed FVA testing with natural blinking without topical anesthesia during a 60 s time interval using an AS-28 FVA measurement system (Kowa, Nagoya, Japan) as described elsewhere [14], in which subjects were allowed to blink naturally without the administration of topical anesthetics. The AS-28 FVA measurement system displays Landolt optotypes automatically for 2 seconds, starting with a size appropriate for the patient's best-corrected visual acuity. The patient uses a joystick to indicate the orientation of each Landolt ring, and the size is decreased automatically when the answer is correct. If the response is incorrect or no response occurs within 2 seconds, a larger optotype is presented. The test lasts for 60 seconds, and the system can measure visual acuity from 40/20 to 20/2000. After instruction from trained medical staff, patients performed a practice test. If a patient had difficulty in taking a test, he/she repeated the same test until the test was performed smoothly. Then, another trial was performed for the study. In this study, two parameters were compared before and after surgery. First, FVA was defined as the average of the visual acuities measured during a 60 s interval. To allow comparisons of changes in visual acuity over time, another parameter, the visual maintenance ratio (VMR), was determined as an objective index calculated by the logMAR values of the FVA scores over the time frame for testing divided by the logMAR baseline visual acuity score. The VMR formula is as follows: VMR = (lowest logMAR VA score−FVA at 60 s)/(lowest logMAR VA score−baseline VA). The VMR is considered to be adequate for statistically comparing groups with different baseline visual acuities [14].

### 2.9. Study Protocol

After enrollment, patients underwent general ophthalmic examinations before and after surgery. A TBUT examination, tear meniscus evaluation, corneal erosion evaluation, tear interferometry test, and FVA test were performed 1–3 days before and 4 weeks after the surgery.

### 2.10. Statistical Analysis

Data were analyzed using the JMP 12.0 software program (SAS Institute, Cary, NC), and the results are presented as the means ± standard deviation (SD). Differences in the results were determined using a paired *t*-test and contingency table analysis. *P* values <0.05 were considered significant.

## 3. Results

### 3.1. Patient Demographics

Thirty eyes of 22 patients satisfied the inclusion criteria and were included in the study protocol. The demographic characteristics of these patients are shown in Table 1. Eight males and 14 females were included. The mean age was 72.2 ± 7.9 years. Primary open-angle glaucoma was the most common type of glaucoma (60% of patients), followed by pseudoexfoliation glaucoma and other types of glaucoma. The total number of eyedrop types and the number of ocular hypotensive eyedrop types used preoperatively were 3.4 ± 0.8 and 3.2 ± 0.7, respectively.

Trabectome surgery significantly improved visual acuity from 0.104 ± 0.179 logMAR to 0.038 ± 0.123 logMAR (*P* < 0.001). Trabectome surgery also significantly reduced IOP from 20.3 ± 5.2 to 15.0 ± 3.3 mmHg (*P* < 0.001) and the number of types of ocular hypotensive eyedrops from 3.2 ± 0.7 to 1.1 ± 0.7 types (*P* < 0.001).

### 3.2. Changes in Ocular Surface Conditions

**Figure 1:**
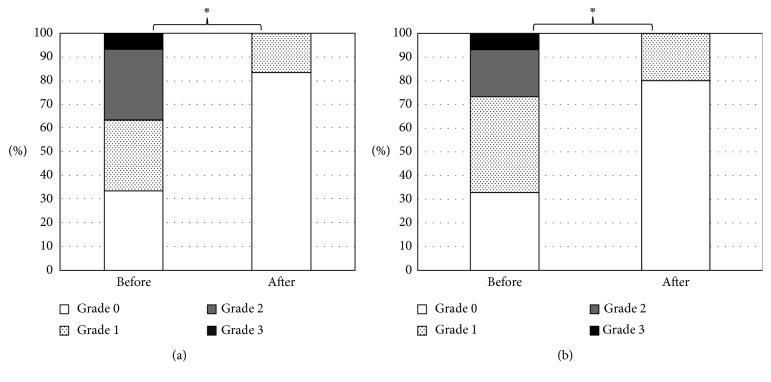
Changes in corneal epithelial damage. Changes in distribution of grades of corneal epithelial damage in area (a) and density (b) before and after Trabectome surgery. ^*∗*^ *P* < 0.001, contingency table analysis.

**Figure 2:**
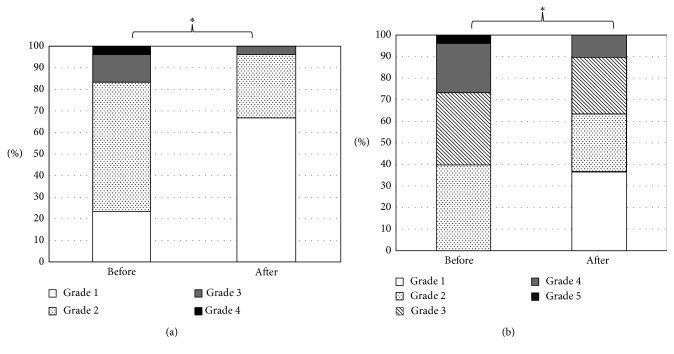
Changes in tear film status. Changes in distribution of tear film spreading grade (a) and tear interferometry grade (b) before and after Trabectome surgery. ^*∗*^ *P* < 0.05, contingency table analysis.

Table 2 shows changes in ocular surface conditions. The TBUT and tear meniscus height were very similar before and after the surgery. The surgery significantly improved corneal erosion (*P* < 0.001) (Figures 1(a) and 1(b)), the tear spreading grade (*P*=0.03) (Figure 2(a)), and the tear interferometry grade (*P*=0.01) (Figure 2(b)). A representative case is shown in Supplementary Videos 1 and 2. He was a 72-year-old male patient with primary open-angle glaucoma (POAG). His tear interferometry grades before Trabectome and after Trabectome were grade 3 and grade 1, respectively.

### 3.3. Functional Visual Acuity Changes

**Figure 3:**
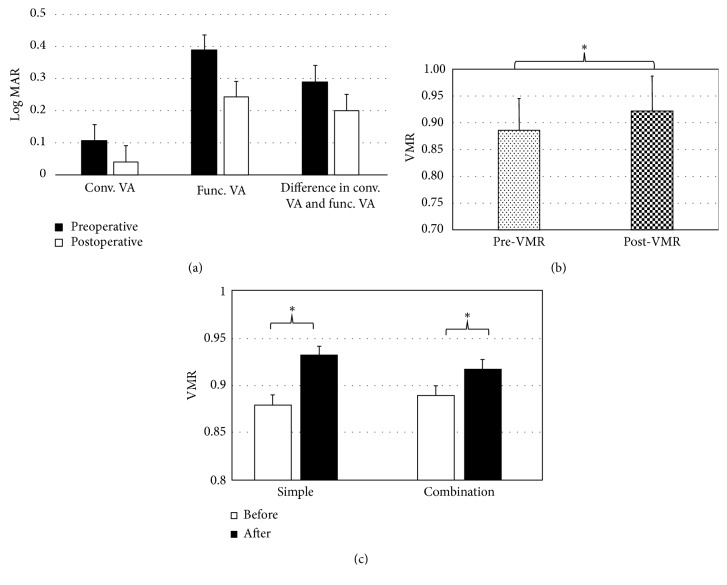
Changes in visual acuity. Comparison of conventional visual acuity, functional visual acuity, and the difference in conventional visual acuity and functional visual acuity between preoperative and postoperative conditions (a); change in the VMR after Trabectome surgery (b); and comparison of changes in the VMR between Trabectome surgery only and Trabectome surgery with phacoemulsification with intraocular lens insertion (c). ^*∗*^ *P* < 0.05, paired *t*-test; conv., conventional; func., functional; VA: visual acuity; MAR: minimum angle resolution; VMR: visual maintenance ratio.

Figure 3(a) shows changes in conventional and FVA after Trabectome surgery. The mean conventional visual acuity significantly improved from 0.104 ± 0.179 logMAR to 0.038 ± 0.123 (*P*=0.01) logMAR, and the mean FVA also significantly improved from 0.340 ± 0.250 logMAR to 0.241 ± 0.244 logMAR (*P*=0.001). The mean FVA was significantly worse than the mean conventional visual acuity at the preoperative and postoperative examinations (*P* < 0.001). The difference in conventional visual acuity before surgery was 0.29 ± 0.270 logMAR, while that after surgery was 0.20 ± 0.230 logMAR, which was a significant difference (*P*=0.03). Trabectome significantly improved the difference in conventional visual acuity and FVA. Trabectome surgery significantly improved the VMR from 0.88 ± 0.06 to 0.92 ± 0.06 among all patients (*P*=0.003) (Figure 3(b)). To eliminate the effect of cataract surgery on the results, we compared changes in the conventional logMAR and VMR between eyes that underwent the Trabectome surgery only and those that underwent the Trabectome surgery combined with cataract surgery. The combined group showed a significant improvement from the presurgical conventional logMAR value to the postsurgical conventional logMAR value (from 0.145 ± 0.184 to 0.054 ± 0.135) (*P*=0.01), while the presurgical and postsurgical conventional logMAR values of the Trabectome surgery-alone group were −0.018 ± 0.050 and −0.008 ± 0.045, respectively, (*P*=0.29). In contrast, both eyes with Trabectome surgery only and those with Trabectome with cataract surgery showed a significant improvement in VMR from 0.88 ± 0.06 to 0.93 ± 0.06 (*P*=0.04) and from 0.89 ± 0.06 to 0.92 ± 0.07 (*P*=0.04), respectively. Both groups showed significant improvements in the VMR of a similar magnitude (Figure 3(c)). A representative case is shown in Supplementary Figure 3. She was a 74-year-old female patient with POAG. Trabectome surgery alleviated the decrease in visual acuity during the test period.

## 4. Discussion

The current study revealed a new aspect of ab interno trabeculotomy using the Trabectome system in addition to that of IOP reduction. The Trabectome surgery improved not only the ocular surface condition, as evidenced by changes in corneal superficial keratitis, tear spreading, and tear interferometry, but also FVA, which may be highly involved in visual function related to daily life. Together, these positive effects of Trabectome surgery could improve the quality of vision.

Instability of the precorneal tear layer causing corneal epithelial damage is related to factors produced by the lacrimal glands and conjunctival goblet cells. In addition, inflammatory mediators may participate in the development of corneal epithelial damage. Ocular hypotensive eyedrops have been reported to deteriorate ocular surface conditions. Rossi et al. reported that the number of medications used, prolonged use of reserved medications, and total BAC exposure were significantly associated with ocular surface disease [15]. Valente et al. also reported that approximately half of glaucoma patients using preserved ocular hypotensive eyedrops showed symptoms of tear film dysfunction and that ocular surface damage seemed to be greater in patients using more than two medications [16]. Lee et al. reported that compared to normal controls, chronically medicated glaucoma patients were more likely to have an increase in tear film osmolarity, which commonly results in dry eye symptoms [17]. Approximately two-thirds of the enrolled eyes in the current study showed a corneal epithelial defect upon enrollment in the study, which is consistent with the previous reports.

Since the tear meniscus height and TBUT did not show any significant change, the role of superficial punctate keratitis, or SPK, improvement might not be due to an increase in tear volume but to alleviation of drug-induced cytotoxicity and/or tear film formation. Chen et al. reported that commercial latanoprost, travoprost, and bimatoprost damaged the corneal epithelium by breaking down the barrier integrity, cell junction, and cytoskeleton but did not affect aqueous tear production or the TBUT [18], which is consistent with the current results. Lee et al. also reported no significant difference in the TBUT and Schirmer's test between chronically medicated patients and patients who underwent trabeculectomy [17]. However, Villani et al. presented a controversial report that showed that the use of preserved ocular hypotensive eyedrops reduced the TBUT [19]. Taken together, ocular hypotensive eyedrops may damage the ocular surface either through direct action on the corneal epithelial cells or by reducing precorneal tear film stability.

Trabectome surgery significantly reduced the number of different types of ocular hypotensive eyedrops used by patients, which may be related to improvement in the damage of the ocular surface. Zhang et al. reported that pilocarpine and timolol have direct effects on human meibomian gland epithelial cells that may influence their morphology, survival, and proliferative capacity [20]. Arita et al. reported that long-term use of antiglaucoma eyedrops was associated with alterations in meibomian gland morphology and function [21]. The number of eyedrop types containing BAC was also significantly reduced by Trabectome surgery (Table 2). BAC contained in eyedrops has been known to possibly damage the ocular surface [22–24]. BAC-preserved ophthalmic formulations could induce acute cytotoxic effects even during a clinically relevant exposure time [24]. Previous papers have reported that compared to preserved eyedrops, preservative-free eyedrops are significantly less associated with ocular symptoms and signs of irritation [22, 23]. Taken together, ocular surface damage may be induced by the additive effect of ocular hypotensive reagents and BAC, although it is impossible to know the exact cause of ocular surface damage.

All patients used antibacterial eyedrops and/or nonsteroidal anti-inflammatory eyedrops at the postoperative examination, which may have influenced the current results. Ayaki et al. reported that ophthalmic antibiotic solutions damaged the corneal epithelium [25], but Price et al. reported that use of ophthalmic solutions containing 0.3% gatifloxacin or 0.5% moxifloxacin did not result in clinically significant epithelial toxicity in healthy human corneas [26]. It is not possible to conclude that ocular hypotensive eyedrops exert a more toxic effect on the ocular surface than other eyedrops because the number of eyedrop types used by patients was not the same between the preoperative and postoperative condition. However, it should be noted that a reduction in the number of ocular hypotensive eyedrop types used may alleviate ocular surface damage.

In the current study, conventional visual acuity and FVA were improved in the eyes that underwent Trabectome surgery combined with cataract surgery; however, the eyes that underwent Trabectome surgery alone did not show any change in conventional visual acuity. In contrast, Trabectome surgery resulted in improvement in the VMR both in eyes treated with Trabectome surgery alone and in those treated with Trabectome surgery combined with cataract surgery. Interestingly, the magnitude of improvement in the VMR was similar between these two groups. The VMR is proposed as a parameter that can be used to compare FVA among eyes with different conventional visual acuity [7, 8]. Patients who have an unstable tear film showed a decreased VMR [8], which reflects the performance ability of specific daily activities that involve visual tasks.

Currently, medical therapy has been considered the first choice for the care for patients with glaucoma. The number of ocular hypotensive eyedrop types used by patients has been increased. Indeed, the previous studies showed an average of approximately 2 types of ocular hypotensive eyedrops being used by patients [1]. Medical therapy has some drawbacks, such as poor adherence to the drug regimen, adverse effects, and low persistency. Ocular hypotensive eyedrops have been reported to induce an adverse effect when the number of different types of ocular hypotensive eyedrops is increased and the prescribing period is prolonged [15]. Since glaucoma is a lifelong disease, a safe and stable treatment regimen is required. Eliminating ocular hypotensive eyedrops by adopting surgeries including the Trabectome procedure could be an option from the view point of ocular safety and quality of vision, in addition to IOP control.

This study has some limitations. The number of enrolled patients was relatively small, and the observation period was short. We evaluated the ocular surface condition and FVA only once after the surgery. To confirm the current results, additional studies utilizing a larger sample size and multiple tests with a long follow-up period after the surgery should be conducted. It is unclear whether Trabectome surgery itself improved ocular surface condition and FVA. The reduction in hypotensive ophthalmic solutions may have played a role in the current results. It has been reported that mitomycin C-augmented trabeculectomy deteriorated ocular surface condition, which was mainly due to increased irregularity of ocular surface configuration and mitomycin C toxicity. MIGS, including Trabectome, may alleviate ocular surface damage, resulting in improved ocular surface condition and visual function. It is necessary to investigate the effects of other MIGS on the ocular surface and visual function. The learning effect for performing the FVA test could not be completely eliminated, although participating patients repeated the FVA test until becoming sufficiently familiar with it that they could perform the test for the study; the reproducibility of this system has been confirmed previously [14]. Masked examiners independently evaluated the ocular surface conditions; however, the accuracy of the evaluations may not be completely accurate because these evaluation methods included subjective classifications.

In conclusion, applying Trabectome surgery may be beneficial not only for IOP reduction but also for improving ocular surface conditions and visual acuity, which may contribute to improvement in the quality of vision. Although it is unclear whether the ocular hypotonic reagent or BAC play a main role in ocular hypotensive eyedrop-related ocular complications, ophthalmologists should consider reducing the number of different types of ocular hypotonic eyedrops used and/or the BAC concentration as much as possible. Introduction of minimally invasive glaucoma surgeries, including the Trabectome approach, may be a good option for improving the quality of vision, although the current results should be confirmed by further studies with longer observation periods and larger sample sizes.

## Figures and Tables

**Table 1:** Demographics: comparison of general ophthalmic status before and after the operation (Table 2).

Number of subjects	30 eyes/22 subjects
Male : female	8 : 14
Age	72.2 ± 7.9 yrs
Type of glaucoma	
POAG	18 eyes
PEX	5 eyes
Others	7 eyes
Alone versus combined	8 versus 22
Preoperative VA (logMAR)	0.104 ± 0.179
HFA MD (program 24-2)	−12.7 ± 7.6 dB
mpNFLT	60.3 ± 10.4 *μ*M
Pre-IOP	20.3 ± 5.2 mmHg
Refractive error	−3.2 ± 4.7 diopter
Phakia versus pseudophakia	28 versus 2
Systemic disease	
CKD	2
Basedow	1
HT	1
Total number of eyedrops	3.4 ± 0.8
Number of ocular hypotensive eyedrops	3.2 ± 0.7

POAG: primary open-angle glaucoma; PEX: pseudoexfoliation glaucoma; MAR: minimum angle resolution; HFA: Humphrey field analyzer; MD: mean deviation; mpNFLT: mean peripapillary nerve fiber layer thickness; IOP: intraocular pressure; CKD: chronic kidney disease; HT: hypertension.

**Table 2:** Comparison between preoperative and postoperative parameters.

	Preoperative	Postoperative
Total number of eyedrops	3.4 ± 0.8	2.5 ± 1.2
Number of ocular hypotensive eyedrops	3.2 ± 0.7	1.1 ± 0.7
Number of BAC-contained eyedrops	3.3 ± 0.9	1.2 ± 0.7
IOP (mmHg)	20.3 ± 5.2	15.0 ± 3.3
LogMAR	0.104 ± 0.179	0.038 ± 0.123
Tear meniscus height (mm)	0.29 ± 0.18	0.30 ± 0.17
TBUT (sec)	6.5 ± 1.0	6.5 ± 1.0

IOP: intraocular pressure; MAR: minimum angle resolution; TBUT: tear breakup time.

## Data Availability

The data used to support the findings of this study are available from the corresponding author upon request.

## Supplementary Materials

Supplementary 1 Figure 1: representative images of the lipid layer interference pattern (modified from reports of Yokoi et al. [12, 13]).

Supplementary 2 Figure 2: representative images of the tear film spreading patterns (modified from reports of Yokoi et al. [12, 13]).

Supplementary 3 Figure 3: a representative case indicating changes in functional visual acuity after Trabectome surgery.

Supplementary 4 Video 1: a representative case indicating changes in the lipid layer interference pattern before Trabectome.

Supplementary 5 Video 2: a representative case indicating changes in the lipid layer interference pattern after Trabectome.
