# Supplementary figures and images for: Reduction in Ocular Hypotensive Eyedrops by Ab Interno Trabeculotomy Improves Not Only Ocular Surface Condition But Also Quality of Vision
Source: J Ophthalmol. 2018 Jun 21;2018:8165476. doi: 10.1155/2018/8165476 (PMC6032976; doi:10.1155/2018/8165476)

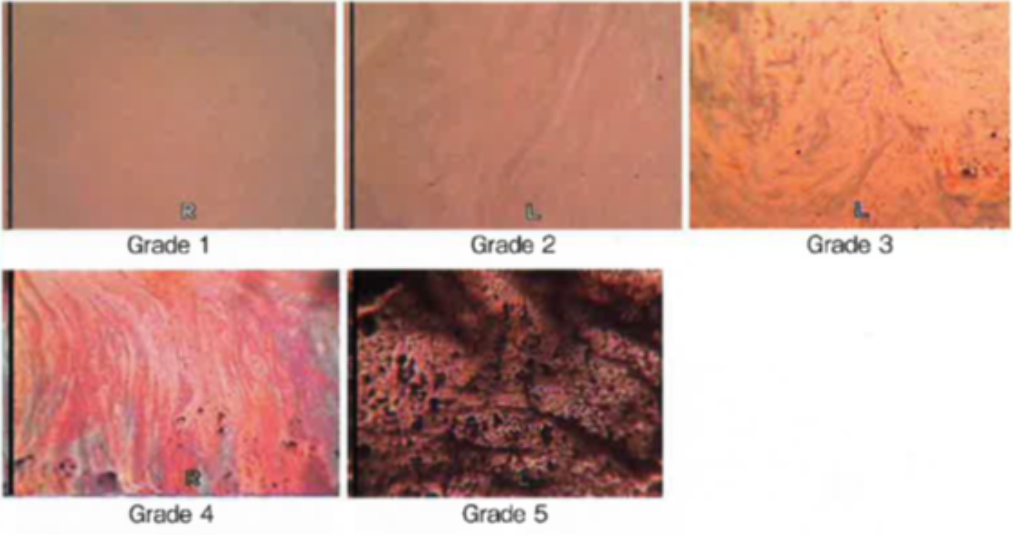

Supplement: Supplementary 1 — Figure 1: representative images of the lipid layer interference pattern (modified from reports of Yokoi et al. [12, 13]). [file 8165476.f1.tif]

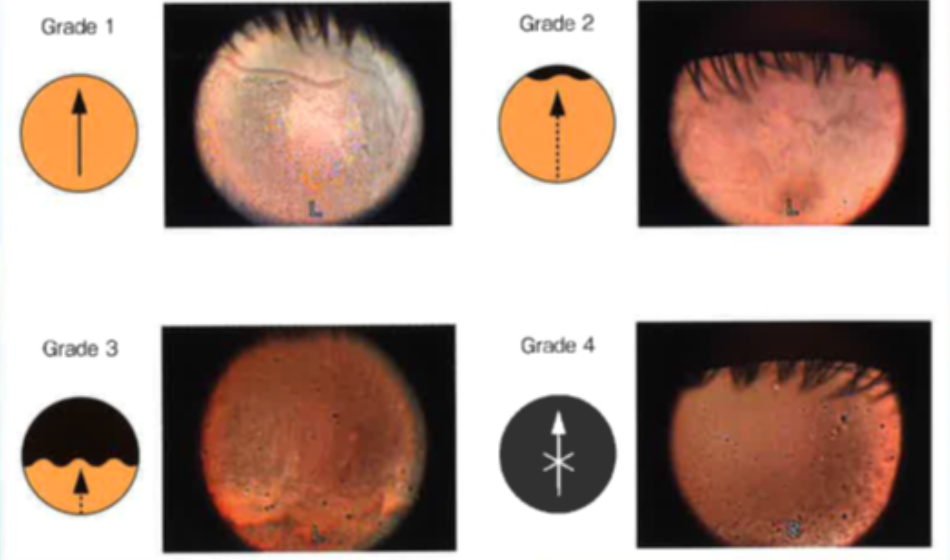

Supplement: Supplementary 2 — Figure 2: representative images of the tear film spreading patterns (modified from reports of Yokoi et al. [12, 13]). [file 8165476.f2.tif]

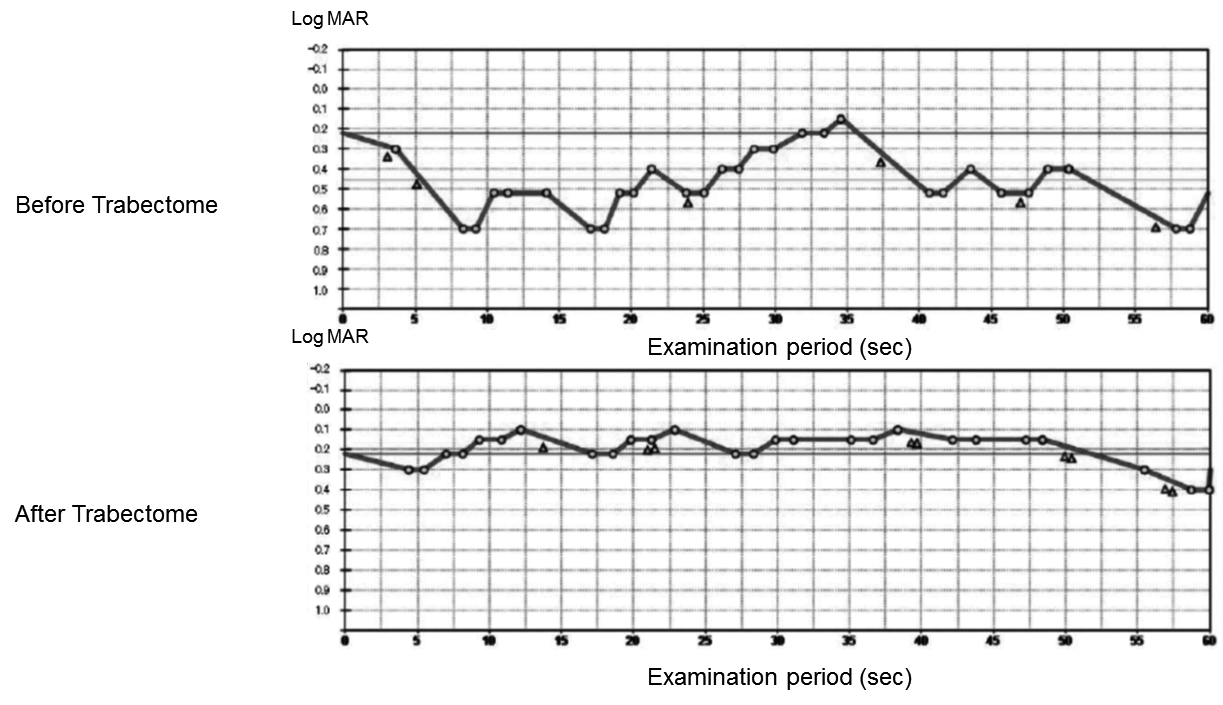

Supplement: Supplementary 3 — Figure 3: a representative case indicating changes in functional visual acuity after Trabectome surgery. [file 8165476.f3.tif]
